# Automated analysis and detection of abnormalities in transaxial anatomical cardiovascular magnetic resonance images: a proof of concept study with potential to optimize image acquisition

**DOI:** 10.1007/s10554-020-02050-w

**Published:** 2020-10-29

**Authors:** James P. Howard, Sameer Zaman, Aaraby Ragavan, Kerry Hall, Greg Leonard, Sharon Sutanto, Vijay Ramadoss, Yousuf Razvi, Nick F. Linton, Anil Bharath, Matthew Shun-Shin, Daniel Rueckert, Darrel Francis, Graham Cole

**Affiliations:** Department of Computing, National Heart and Lung Institute, Imperial College London, Imperial College Healthcare NHS Trust, London, UK

**Keywords:** Artificial intelligence, Machine learning, Cardiac magnetic resonance imaging, Neural networks

## Abstract

The large number of available MRI sequences means patients cannot realistically undergo them all, so the range of sequences to be acquired during a scan are protocolled based on clinical details. Adapting this to unexpected findings identified early on in the scan requires experience and vigilance. We investigated whether deep learning of the images acquired in the first few minutes of a scan could provide an automated early alert of abnormal features. Anatomy sequences from 375 CMR scans were used as a training set. From these, we annotated 1500 individual slices and used these to train a convolutional neural network to perform automatic segmentation of the cardiac chambers, great vessels and any pleural effusions. 200 scans were used as a testing set. The system then assembled a 3D model of the thorax from which it made clinical measurements to identify important abnormalities. The system was successful in segmenting the anatomy slices (Dice 0.910) and identified multiple features which may guide further image acquisition. Diagnostic accuracy was 90.5% and 85.5% for left and right ventricular dilatation, 85% for left ventricular hypertrophy and 94.4% for ascending aorta dilatation. The area under ROC curve for diagnosing pleural effusions was 0.91. We present proof-of-concept that a neural network can segment and derive accurate clinical measurements from a 3D model of the thorax made from transaxial anatomy images acquired in the first few minutes of a scan. This early information could lead to dynamic adaptive scanning protocols, and by focusing scanner time appropriately and prioritizing cases for supervision and early reporting, improve patient experience and efficiency.

**Electronic supplementary material:**

The online version of this article (10.1007/s10554-020-02050-w) contains supplementary material, which is available to authorized users.

## Background

CMR offers limitless scan planes and a large range of different sequences to characterize the heart in different ways, but this means that the acquisition process must be selective because not every patient can have every imaging plane scanned with every sequence. Efficient clinical practice therefore operates through standardized “protocols”, which list the sequences that are most likely to generate images which confirm or refute important diagnoses for particular clinical scenarios [1].

However, sometimes the earliest images acquired during the scan itself reveal unexpected findings which may (a) indicate that some previously protocolled images are no longer required or (b) indicate that further images should be acquired to confirm or refute a previously unsuspected diagnosis. Some sequences are best interpreted before gadolinium contrast has been given, meaning that the opportunity to use them may be lost if the requirement for them is not spotted early.

In recent years, deep learning using neural networks has shown increasing performance in the classification and segmentation of medical imaging data [2]. Recent work has shown similar precision to human techniques at tasks such as left ventricular (LV) segmentation [3–5]. These approaches, however, have focused on the segmentation of high-quality LV-dedicated sequences with ideal scan plane orientation.

Within the first couple of minutes of a CMR scan, a series of transaxial images are routinely acquired, commonly termed the “anatomy” sequences. In this study we investigate whether deep learning methods could analyze these early images to extract information that could (a) help a radiographer recognize the need to modify the ongoing protocol, (b) help identify cases that should be prioritized for medical supervision or early reporting.

## Methods

### Data extraction

We extracted 575 sequential CMR scans from 2 manufacturers (Philips Healthcare, Amsterdam, Netherland & Siemens, Erlangen, Germany) performed across 2 centers. Inclusion criteria were adult scans in which a bright-blood transaxial anatomical sequence had been performed and a valid final report which included the following BSA-indexed (suffix *i*) measurements as a minimum: LV end-diastolic volume (LVEDV_i_), LV mass (LVM_i_) and RV end-diastolic volume (RVEDV_i_). We did not exclude scans with artefact, e.g. due to atrial fibrillation or breath-holding difficulties. We also extracted the following measures from the report where present: the diameter of the ascending aorta; the presence of hypertrophic cardiomyopathy (HCM), the presence of dilated cardiomyopathy (DCM) and the presence of pleural effusions.

**Fig. 1:**
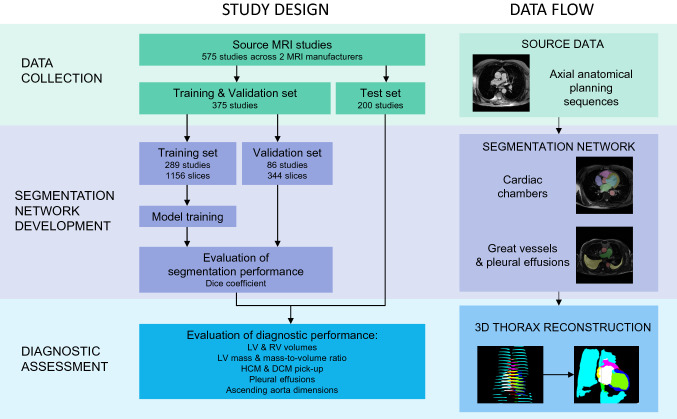
Study design flow chart and data flow. The aim of this study was to use artificial intelligence (AI) to create a 3D model of the thorax for each patient using the anatomy images acquired within the first few minutes of a cardiac MRI scan. This could be used to make measurements and provide useful diagnostic information within minutes of a scan starting. The study comprised 575 studies across 2 manufacturers, which were split into a training and testing set. The training set was used to train an AI which could segment each slice of the anatomy sequence. Then, each scan in the testing set was analyzed to allow creation of a 3D model of a patient’s thorax. This 3D model was then analyzed. 3D = 3-dimensional. DCM = dilated cardiomyopathy. HCM = hypertrophic cardiomyopathy. LV = left ventricle, RV = right ventricle

The CMR scans were then randomly assigned to different datasets, each serving a specific purpose (Fig. 1; Study Design). 200 scans were assigned to the “testing dataset”. This dataset was used to report the final accuracy of the system and was not shown to the neural network at any stage of its training. The remaining 375 scans were assigned to the “training & validation dataset”. This dataset was used to train the neural network. 75% of these scans were used to directly train the neural network (the “training dataset”), and 25% were used to continually appraise the performance of the network during development (the “validation dataset”).

Ethical approval was gained from the Health Regulatory Agency (Integrated Research Application System identifier 243,023).

### Data processing and labelling

The axial anatomical images were isolated from each scan. The acquisition parameters for these sequences are shown in Appendix 1. 1500 slices were then chosen at random, across the training and validation sets and were then labelled by clinicians using custom-designed software to draw around the following anatomical features, if present: ascending aorta, aortic arch, descending aorta, left atrium, left ventricle, pericardial effusion, pleural effusion, right atrium and right ventricle. Each slice was then resampled to 560 by 560 pixels, with zero-padding for non-square acquired images.

### Neural network design and training

The neural network design chosen was an adapted version of the HRNet architecture [6]. Modifications were made so the network could receive single-channel (grayscale) images and output 9 feature maps, corresponding to the possible identities of each pixel (aorta, left atrium, left ventricular wall, left ventricular cavity, pulmonary artery, pleural effusion, right atrium, right ventricular cavity) or a final ‘other’ (background) class. The network was trained until the validation loss plateaued (23 epochs), and the training process was augmented with random rescaling, rotation, shearing and translation. Loss was calculated over batches of 20 images by using the categorical cross-entropy loss function, and weights were updated using the Ranger optimizer (a combination of RAdam [7] and Lookahead [8]) with a learning rate of 0.001. The optimizer, choice of data augmentations and learning rate were tuned with reference to the validation loss. Programming was performed using the Python programming language with the Pytorch framework [9]. Training was performed on 2 GeForce RTX Titan graphical processing units (NVIDIA, Santa Clara, California).

### 3D heart model reconstruction

**Fig. 2:**
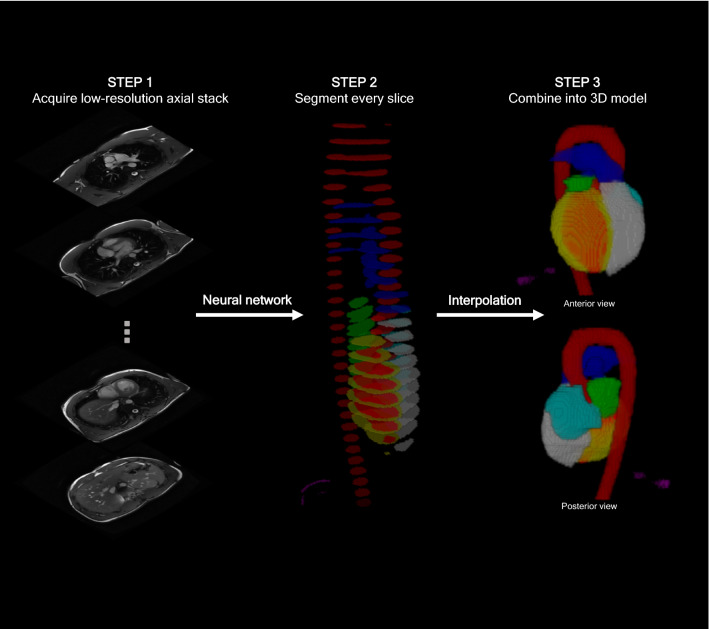
Reconstruction of a 3D heart is possible by segmenting each slice within the axial anatomy stack. The slices can be combined through trilinear interpolation into a 3D model of the cardiac chambers and great arteries. This model can be used to make anatomical measurements, such as end-diastolic volumes and vessel diameters

The segmentation predictions of the neural network were converted into predictions of parameters such as cardiac chamber sizes by analyzing each slice within the axial anatomical planning sequence and then re-assembling them into a 3D heart model (Fig. 2). Specifically, each slice was fed into the neural network to yield a prediction of the identity of each pixel (left ventricle, pleural effusion, etc.). The slices were then resized according to the viewport information embedded in the DICOM file and the thickness of the slice, before combining the stacked series of slices into a 3D model through trilinear interpolation. This model was then analyzed by calculating the volume of each structure present to give a value in ml. These were then scaled for body surface area to yield a value in ml/cm^3^. Finally, these volumes were adjusted to the “final” analyzed volumes by fitting a linear regression model separately for each scanner manufacturer: this allowed a bias to be introduced to account for the different acquisition parameters between the two manufacturers. Models were fitted for estimating LVEDV_i_, RVEDV_i_, LVM_i_, BSA-indexed LV mass-to-volume ratio, and BSA-indexed ascending aortic diameter. This allowed compensation for any systematic error associated with the use of differences in sequences employed by different manufacturers.

For each measure, published BSA-indexed cut-offs were used to classify abnormal cases: LVEDVi > 94 ml/m^2^ for LV dilatation, RVEDVi > 98 ml/m^2^ for RV dilatation, LVMi > 83.5 g/ml for LV hypertrophy [10], and > 0.84 for an abnormal LV mass-to-volume ratio [11].

### Endpoints and statistical analysis

The segmentation performance of the neural network was assessed using the Dice coefficient, defined as:

$$Dice= \frac{2 \cdot TP}{2\cdot TP+FP+FN}$$

where for each class, TP refers to true positives (pixels classified by both the network and expert as of that class), FP as false positives (pixels classified by the network as that class, incorrectly) and FN as false negatives (pixels classified by the network as not that class, incorrectly).

The diagnostic performance of the network was reported for continuous outcomes using Pearson’s *r*, along with the accuracy, sensitivity and specificity. Cohen’s Kappa (κ) was also reported as a measure of accuracy resistant to class imbalance. For categorical outcomes, we reported the area under the curve (AUC) for the receiver operating characteristic (ROC), along with the accuracy and Cohen’s Kappa for the optimal cut-off. Statistical analysis was performed using R software (R Foundation, Vienna, Austria) [12].

## Results

### Dataset

The baseline characteristics across the training & validation dataset and the testing dataset are shown in Table 1.

**Table 1:** Characteristics of the included studies in the training and testing sets. Values are mean (standard deviation) for continuous variables, and count (percentage) for categorical variables

	Training and validation set	Testing set
Number of studies/patients	375	200
*Manufacturer*		
Siemens	221	125
Philips	154	75
Male	229 (61.1)	115 (57.5)
Age (years)	55.7 (17.8)	56.7 (17.5)
LVEF (%)	55.6 (17.5)	55.7 (17.8)
LVEDV_i_ (ml/m^2^)	81.4 (34.6)	81.0 (38.1)
RVEDV_i_ (ml/m^2^)	80.1 (25.9)	78.8 (26.6)
LVM_i_ (g/m^2^)	77.6 (30.8)	76.4 (33.2)
Ascending aorta (mm/m^2^)	17.1 (4.2)	16.8 (4.1)
HCM	25 (6.7)	10 (5.0)
DCM	22 (5.9)	10 (5.0)
Pleural effusions	88 (23.5)	42 (21.0)

*LVEF* left ventricular ejection fraction, *LVEDV*^*i*^ Body-surface area-indexed left ventricular end-diastolic volumes, *RVEDV*^*i*^ Body-surface area-indexed right ventricular end-diastolic volumes, *LVM*^*i*^ Body-surface area-indexed right ventricular end-diastolic volumes, *HCM* hypertrophic cardiomyopathy, *DCM* dilated cardiomyopathy

1500 slices underwent segmentation labelling, 1156 of which were within the training set and 344 within the testing set. Table 2 outlines the contents of the slices.

**Table 2:** Slices included in the training and testing sets

Feature	True presence within a slice		Network performance (Dice coefficient)
	Training set (n = 1156)	Validation set (n = 344)	
Aorta	1059 (91.6)	316 (91.9)	0.929
Left atrium	404 (34.9)	116 (33.7)	0.925
LV cavity	558 (48.3)	151 (44.1)	0.809
LV wall	587 (50.8)	167 (48.6)	0.884
Pulmonary artery	182 (15.8)	65 (18.9)	0.907
Pleural effusion	347 (30.9)	128 (37.2)	0.890
Right atrium	481 (41.6)	139 (40.4)	0.924
RV cavity	616 (53.3)	178 (51.7)	0.910
Background	1156 (100.0)	344 (100.0)	0.995

Values are n (%). The Dice coefficient reflects the accuracy of the neural network on the validation set

### Segmentation performance

The mean Dice coefficient across all categories for the testing set was 0.910. The category-wise Dice coefficients are shown in Table 2 and ranged between 0.809 for the LV cavity to 0.929 for the aorta.

**Fig. 3:**
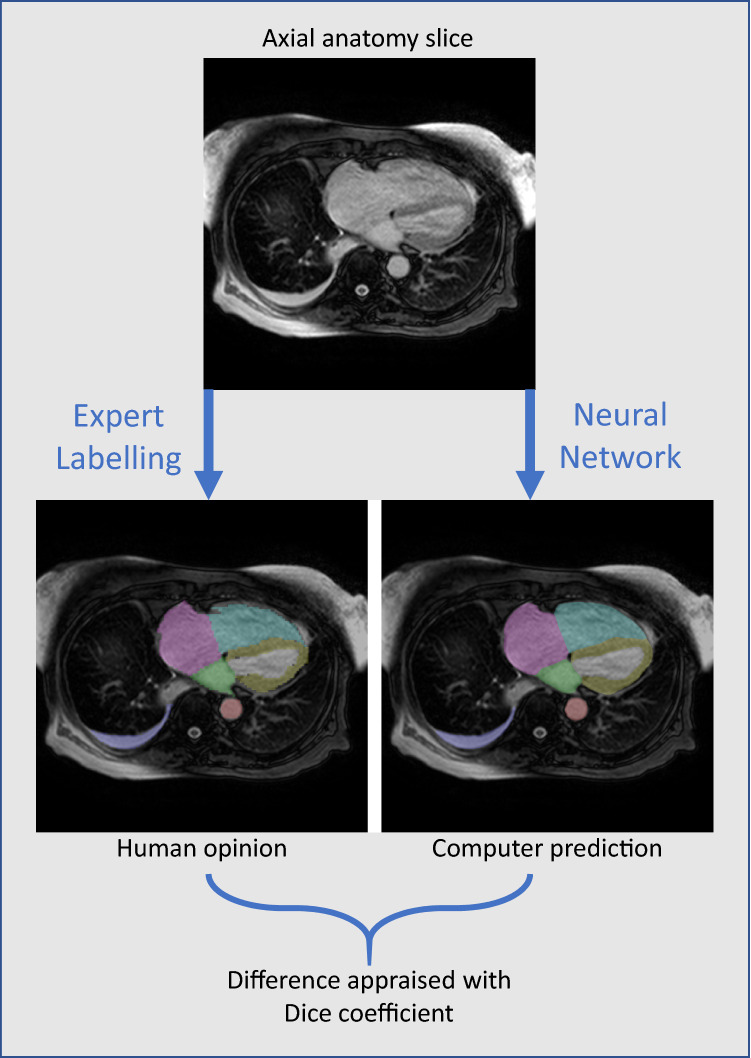
A Human label and the AI’s segmentation corresponding prediction. The figure showed a comparison between the human labels of a particular axial anatomical planning slice, and those predicted by the network for that slice

Examples of the human-supplied labels and the predictions of the neural network are shown in Fig. 3. In contrast, the slice where the neural network made the greatest segmentation error is shown in the Appendix.

### Diagnostic model performance

By combining every slice from a scan’s axial anatomic planning sequence, we were able to construct a 3D model of a patient’s thorax (Fig. 2) from which calculations could be made of chamber size but also for presence of abnormal features, such as pleural effusions.

**Fig. 4:**
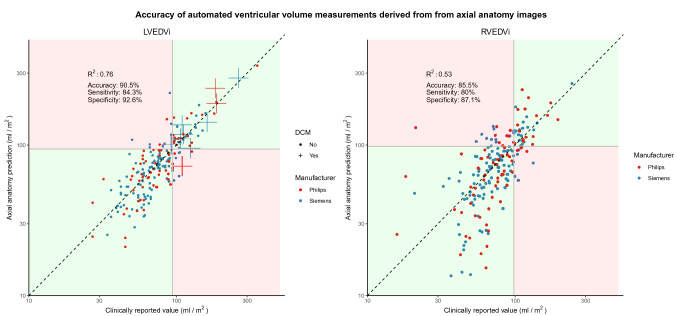
The relationship between the neural network’s chamber size predictions versus those extracted from the final report. Predictions are shown for BSA-indexed left ventricular end-diastolic volume (LVEDVi) and BSA-indexed right ventricular end-diastolic volume (RVEDVi). Green zones and red zones symbolize areas of the plot where the neural network agrees and disagrees with the values from the full scan, respectively. Patients with known dilated cardiomyopathy (DCM) are shown as crosses on the LVEDVi plot

BSA-indexed left ventricular end diastolic volume (LVEDVi) predicted by the network on the testing set correlated with the measures from the final report (*R*^*2*^ = 0.76, p < 0.0001, Fig. 4). The network was 90.5% accurate in identifying LV dilatation (κ = 0.75), with a corresponding sensitivity of 84.3% and specificity of 92.6%. Within the testing dataset, 10 studies were from patients with dilated cardiomyopathy, and the network correctly identified 9 (90%) of these as abnormal (95% CI 59.6% to 98.2%).

BSA-indexed right ventricular end diastolic volume (RVEDVi) predicted by the network on the testing set correlated with the measures from the final report (*R*^*2*^ = 0.53, p < 0.0001, Fig. 4). The network was 85.5% accurate in identifying RV dilatation (κ = 0.62), with a corresponding sensitivity of 80% and specificity of 87.1%.

**Fig. 5:**
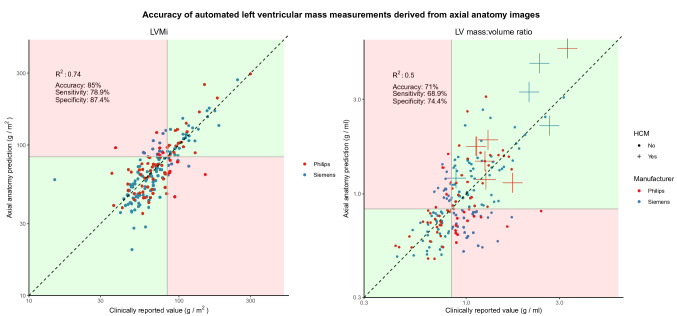
The relationship between the neural network’s left ventricular mass predictions versus those extracted from the final report. Predictions are shown for BSA-indexed left ventricular mass (LVMi) and the left ventricular mass:volume ratio, where volume is derived from the neural network’s prediction of the left ventricular end diastolic volume. Green zones and red zones symbolize areas of the plot where the neural network agrees and disagrees with the values from the full scan, respectively. Patients with known hypertrophic cardiomyopathy (HCM) are shown as crosses on the LVMi plot

BSA-indexed left ventricular mass (LVMi) predicted by the network on the testing set correlated with the measures from the final report (*R*^*2*^ = 0.74, p < 0.0001, Fig. 5). The network was 85% accurate in identifying LV hypertrophy (κ = 0.64), with a corresponding sensitivity of 78.9% and specificity of 87.4%. LV mass:volume ratio predicted by the network on the testing set correlated with the measures from the final report (*R*^*2*^ = 0.5, p < 0.0001, Fig. 5). The network was 71% accurate in identifying a raised LV mass:volume ratio (κ = 0.41), with a corresponding sensitivity of 68.9% and specificity of 74.4%. Within the testing dataset, 10 studies were from patients with hypertrophic cardiomyopathy, and the network correctly identified all (100%) of these as abnormal (95% CI 72.3% to 100%).

**Fig. 6:**
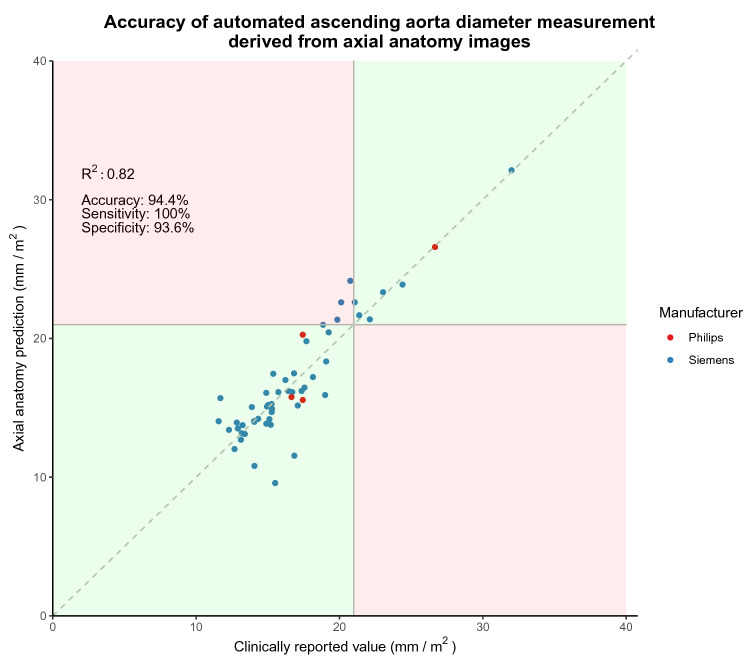
The relationship between the neural network’s BSA-indexed ascending aortic diameter predictions versus those extracted from the final report. Green zones and red zones symbolize areas of the plot where the neural network agrees and disagrees with the values from the full scan, respectively

Only 54 of the 200 testing dataset cases provided ascending aorta diameters. BSA-indexed ascending aorta diameters predicted by the network on the testing set correlated with the measures from the final report (*R*^*2*^ = 0.82, p < 0.0001, Fig. 6). The network was 94.4% accurate in identifying ascending aortic dilatation (κ = 0.79), with a corresponding sensitivity of 100% and specificity of 93.6%.

The ROC curve AUC for diagnosing pleural effusions by quantifying pleural fluid was 0.906 (see online Appendix).

## Discussion

This study is a proof-of-concept, that accurate important diagnostic information can be derived from axial anatomy images obtained at the start of a CMR scan. These results are consistent across two different scanner manufacturers. This system could allow technicians performing the scan to be signposted to unexpected pathology, help direct optimized image acquisition for the remainder of the scan, prioritize supervision of scans by reporters, and prioritize scans for urgent reporting.

Earlier & automated diagnosis – a comparison with previous approaches.

Neural networks are now rivalling and surpassing humans for cardiac chamber segmentation and quantification [3, 4]; a situation in which images are acquired in a dedicated conventional manner for all patients. However, the aim of our study was different in two ways.

First, the network in this study has the potential to identify extra-cardiac diagnoses such as aortic dilatation and pleural effusions. Being able to identify findings may allow an adaptive approach to scanning protocols which avoids recalling patients for additional images and even gives technicians performing the scan reassurance that additional images are not required.

Second, previous studies have aimed to improve absolute quantification of chamber size and function and have therefore been trained to process the high-quality cine imaging which reporting clinicians currently use. Such sequences differ from the anatomical stack generated and processed in this paper in several important ways.

First, the dedicated sequences’ scan planes are ideally orientated with respect to the patient’s heart to ensure radial function is within rather than through the plane of imaging and minimizes partial voluming. Second, the dedicated sequences are acquired with less spacing, allowing more voxels per ventricle. Third, the dedicated sequences take considerably longer to acquire: they are acquired over 8–15 s per slice, of which there may be up to 10 slices per sequence [3].

In contrast, the entire axial anatomical sequences can be acquired in 3–4 breath holds. However, they are of relatively large slice thickness, and are orientated axially with no reference to the patient’s heart. Given this, the ability to acquire accurate diagnostic information early on in the scan has the potential to triage the application of further sequences during the scan. For example, patients found to have LVH and pleural effusions on axial anatomy images may have a diagnosis of cardiac amyloid in which pre-contrast T1 maps would be useful [13]. Dedicated aortic imaging may be too laborious for routine use on each patient but could be reliably targeted to those who need it by our work.

### Implications for reporting prioritization, supervision and patient safety

A system providing automated diagnosis during the earliest stages of a cardiac MRI scan would not only be useful for ensuring scans are correctly protocolled but would also allow physicians to prioritize the supervision and reporting of those scans most likely to be abnormal.

Patients with pleural effusions, for example, may have limited tolerance for lying flat in the scanner. Their identification at the earliest stages of could increase vigilance of these more vulnerable patients, and alteration of sequences to allow smaller breath holds, motion-corrected free-breathing sequences and accelerated protocols to minimize scan time.

Scans shown to contain unexpected pathologies may also be flagged for expedited review and reporting by physicians. For example, an outpatient screening CMR scan in a low risk patient might be identified as demonstrating unexpected marked LV dilatation with pleural effusions. Such patients at risk of decompensation and could be identified for early reporting and follow-up.

### Study limitations

The neural network described in this study is not 100% accurate. Even the most accurate measurement (ascending aortic diameter) is only 94% accurate on our dataset. However, the correlation between the neural network’s predictions and the true measurements for the measures examined ensure that the extreme biological values associated with disease are more consistently correctly identified by the network (100% of hypertrophic cardiomyopathy cases, 90% of dilated cardiomyopathy cases).

It is difficult to ascertain to what extent the errors in the neural network’s predictions are due to inaccurate segmentation by the network, versus limitations inherent to estimating volumes using anatomy sequence slices. The latter could be estimated by calculating volumes using expert labels across the testing dataset, although this would require every myocardial slice in these data to be labelled. Whilst this dataset would be many times larger than the current dataset used to train the network, we hope to address this question in the future.

Furthermore, previous studies have shown the coefficient of variation is over 10% for estimating left ventricular volumes, even when assessed by the same doctor in the same patient using dedicated left ventricular sequences [3]. In this study, we have compared the performance of this network against human observers behaving clinically, and therefore this 10% variation inherently sets an upper ceiling on the correlation coefficient obtainable by the network—it cannot correlate with the human observers better than the human observers correlate with themselves.

With all deep learning studies, there is concern that the findings in this study may not generalize to a wider population [14]. This can be due to a phenomenon of “overfitting”, where the neural network is highly accurate at processing images on which it was trained but performs much less well on unseen “real world” examples. To try and mitigate these concerns, we took several approaches. First, the performance is reported on a test set which was only assessed after training the neural network. Second, the dataset we assembled was from two different hospitals across multiple reporting physicians. Third, the dataset comprises scans across both Siemens and Philips scanners, with the correlation plots showing similar accuracies for both manufacturers. Finally, we are releasing the neural network with this manuscript for use online, so that its performance can be assessed by any interested academic or clinician.

## Conclusion

This proof-of-concept study demonstrated that a neural network can accurately reconstruct a 3-dimensional model of the heart and major vessels from transaxial anatomy images acquired in the first few minutes of a CMR study. Our system is able to accurately quantify cardiac chamber size, aortic diameter and presence of pleural effusions. We have made trained neural networks publicly available for use.

## Electronic supplementary material

Below is the link to the electronic supplementary material.

Supplementary file1 (DOCX 17 kb)

Supplementary file2 (PDF 4 kb)

Supplementary file3 (DOCX 206 kb)

## Data Availability

The trained neural network and inference code will be made available on the author’s website at https://james.dev—the source data used to train these networks, however, are not publicly available due to ethical restrictions.

## Abbreviations

3D: Three-dimensional
AUC: Area under curve
CMR: Cardiovascular magnetic resonance
DCM: Dilated cardiomyopathy
HCM: Hypertrophic cardiomyopathy
LV: Left ventricle
LVEF: Left ventricular ejection fraction
LVEDVi: LV end-diastolic volume
LVMi: Left ventricular mass
ROC: Receiver operating characteristic
RVEDVi: Right ventricular end-diastolic volume
