# Supplementary material for: Automated analysis and detection of abnormalities in transaxial anatomical cardiovascular magnetic resonance images: a proof of concept study with potential to optimize image acquisition
Source: Int J Cardiovasc Imaging. 2020 Oct 29;37(3):1033–42. doi: 10.1007/s10554-020-02050-w (PMC7969571; doi:10.1007/s10554-020-02050-w)
Supplement: Supplementary file 1 — Supplementary file1 (DOCX 17 kb) [file 10554_2020_2050_MOESM1_ESM.docx]

# Appendix Table 1.

|  | Siemens | Philips |
| --- | --- | --- |
| Sequence type | TRUFI (GRE) | FFE (GRE) |
| ECG gated | Yes | Yes |
| Trigger delay (ms) | 595 | 276 |
| Field Strength (T) | 1.5 | 1.5 |
| Breath held | Yes | Yes |
| Flip angle (degrees) | 80 | 60 |
| Repetition time echo spacing (ms) | 3.24 | 3.109 |
| Echo time (ms) | 1.39 | 1.555 |
| Imaging frequency (Hz) | 63.67 | 63.9 |
| Rows (pixels) | 256 | 432 |
| Columns (pixels) | 256 | 432 |
| Slice thickness (mm) | 6 | 10 |
| Slice spacing (mm) | 6 | 11 |
| Voxel size (mm; height × width × length) | 6.00 × 1.41 × 1.41 | 10.0 × 0.62 × 0.62 |

**Appendix Table 1.** Anatomical planning sequence parameters by manufacturer. FFE = Fast Field Echo; GRE = Gradient Echo; TRUFI = True Fast Imaging with steady-state free procession.
