# Supplementary figures and images for: Automated analysis and detection of abnormalities in transaxial anatomical cardiovascular magnetic resonance images: a proof of concept study with potential to optimize image acquisition
Source: Int J Cardiovasc Imaging. 2020 Oct 29;37(3):1033–42. doi: 10.1007/s10554-020-02050-w (PMC7969571; doi:10.1007/s10554-020-02050-w)

Pleural effison ROC Curve – AUC 0.906

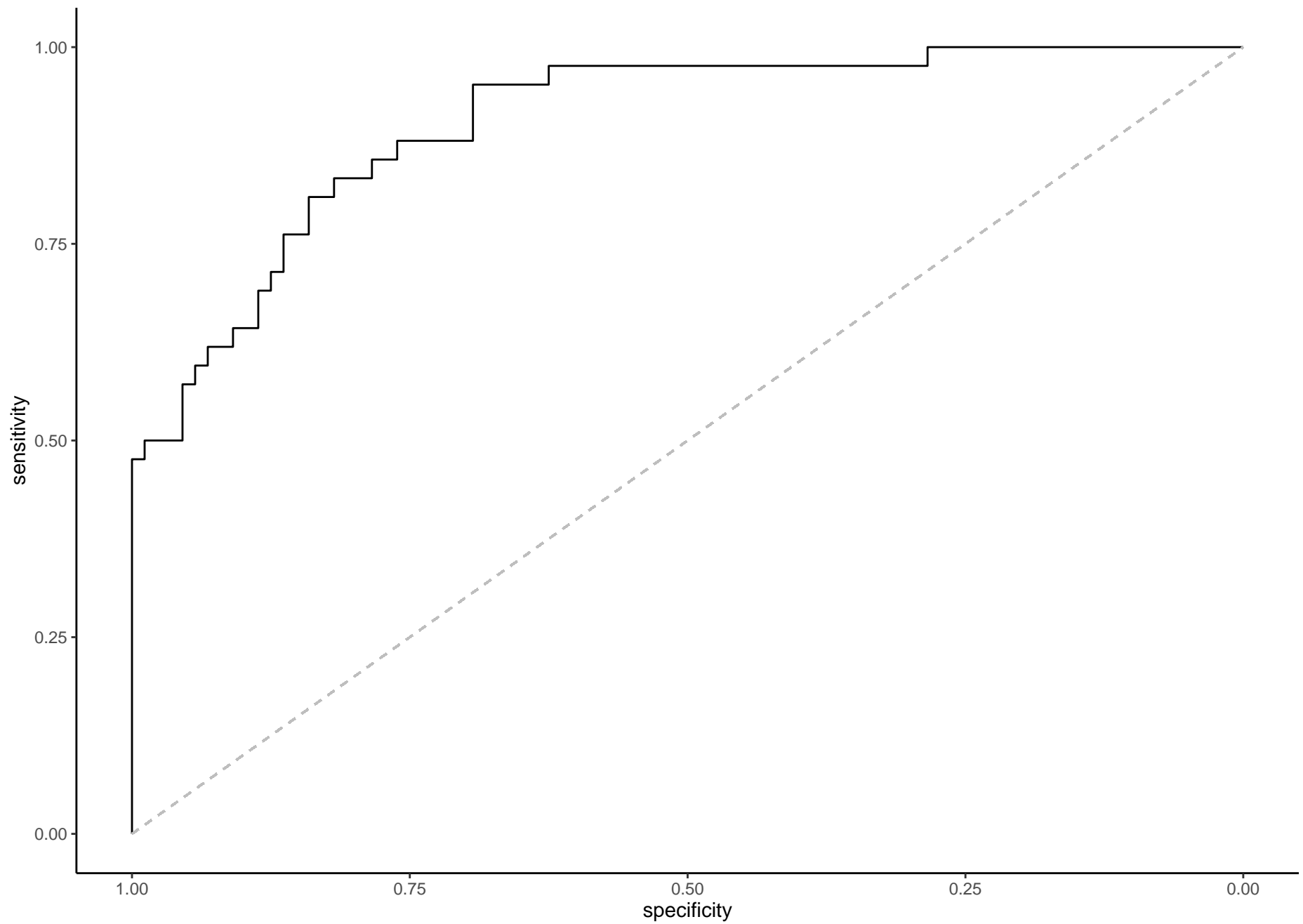

Supplement: Supplementary file 2 — Supplementary file2 (PDF 4 kb) [file 10554_2020_2050_MOESM2_ESM.pdf]
