# Supplementary material for: Automated analysis and detection of abnormalities in transaxial anatomical cardiovascular magnetic resonance images: a proof of concept study with potential to optimize image acquisition
Source: Int J Cardiovasc Imaging. 2020 Oct 29;37(3):1033–42. doi: 10.1007/s10554-020-02050-w (PMC7969571; doi:10.1007/s10554-020-02050-w)
Supplement: Supplementary file 3 — Supplementary file3 (DOCX 206 kb) [file 10554_2020_2050_MOESM3_ESM.docx]

| 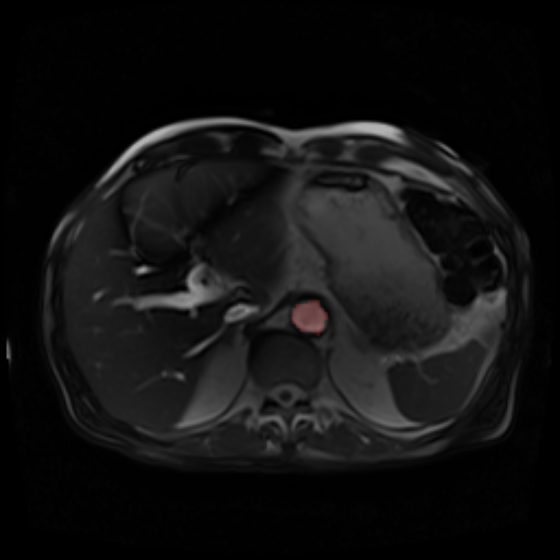 | 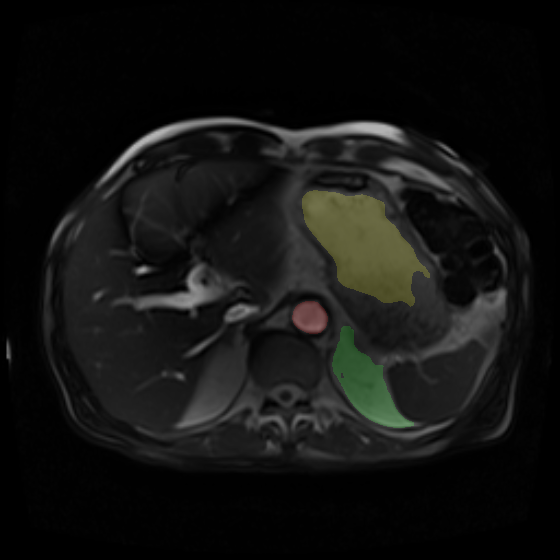 |
| --- | --- |

**Appendix Figure - Example of segmentation failure.** The human-labelled slice is shown on the left, with only the aorta (red) segmented as a region of interest. The neural network (right) has correctly identified the aorta but has made two further errors. First, it believes the roof of the stomach is the inferior LV wall (shaded in yellow). It has also segmented an area of abdominal visceral fat as pleural effusion (green). The latter is particularly surprising, as it has not mistaken the corresponding fat on the patient’s right side as effusion.
